# Sulcal Polymorphisms of the IFC and ACC Contribute to Inhibitory Control Variability in Children and Adults

**DOI:** 10.1523/ENEURO.0197-17.2018

**Published:** 2018-03-08

**Authors:** Cloélia Tissier, Adriano Linzarini, Geneviève Allaire-Duquette, Katell Mevel, Nicolas Poirel, Sonia Dollfus, Olivier Etard, François Orliac, Carole Peyrin, Sylvain Charron, Armin Raznahan, Olivier Houdé, Grégoire Borst, Arnaud Cachia

**Affiliations:** 1 Laboratory for the Psychology of Child Development and Education, Sorbonne, CNRS UMR 8240, Paris, France; 2 Paris Descartes University, Sorbonne Paris Cité, Paris, France; 3 Laboratoire de Recherche en Neuroéducation, Université du Québec à Montréal, Québec, Canada; 4 Institut Universitaire de France, Paris, France; 5 Université de Caen Normandie, Faculté de médecine, Imagerie et Stratégies Thérapeutiques de la Schizophrénie, EA-7466, Caen 14000, France; 6 Centre Hospitalier Universitaire de Caen, Service des Explorations Fonctionnelles du Système Nerveux, Caen 14000, France; 7 Laboratoire de Psychologie et NeuroCognition CNRS UMR 5105, Université Grenoble Alpes, Grenoble F-38000, France; 8 Biomarkers of Brain Development and Disorders, INSERM UMR 894, Center of Psychiatry and Neuroscience, Paris, France; 9 Developmental Neurogenomics Unit, National Institutes of Mental Health, Bethesda, MD 20892

**Keywords:** anterior cingulate cortex, inferior frontal gyrus, inhibitory control, neurodevelopment, sulcation

## Abstract

Inhibitory control (IC) is a core executive function that enables humans to resist habits, temptations, or distractions. IC efficiency in childhood is a strong predictor of academic and professional success later in life. Based on analysis of the sulcal pattern, a qualitative feature of cortex anatomy determined during fetal life and stable during development, we searched for evidence that interindividual differences in IC partly trace back to prenatal processes. Using anatomical magnetic resonance imaging (MRI), we analyzed the sulcal pattern of two key regions of the IC neural network, the dorsal anterior cingulate cortex (ACC) and the inferior frontal cortex (IFC), which limits the inferior frontal gyrus. We found that the sulcal pattern asymmetry of both the ACC and IFC contributes to IC (Stroop score) in children and adults: participants with asymmetrical ACC or IFC sulcal patterns had better IC efficiency than participants with symmetrical ACC or IFC sulcal patterns. Such additive effects of IFC and ACC sulcal patterns on IC efficiency suggest that distinct early neurodevelopmental mechanisms targeting different brain regions likely contribute to IC efficiency. This view shares some analogies with the “common variant–small effect” model in genetics, which states that frequent genetic polymorphisms have small effects but collectively account for a large portion of the variance. Similarly, each sulcal polymorphism has a small but additive effect: IFC and ACC sulcal patterns, respectively, explained 3% and 14% of the variance of the Stroop interference scores.

## Significance Statement

Inhibitory control (IC) is a cognitive function that plays a critical role in the pathophysiology of several psychiatric conditions and in academic and professional success. Using anatomical magnetic resonance imaging (MRI) of healthy children and adults, we found that IC efficiency is constrained by the morphology (sulcal pattern) of two key regions of the neural network underlying IC. Because the sulcal pattern is a morphologic feature of cortical anatomy that is determined during fetal life and stable during development, our findings provide evidence that interindividual differences in IC partly trace back to prenatal processes and that distinct early neurodevelopmental mechanisms targeting different brain regions likely contribute to IC efficiency.

## Introduction

Inhibitory control (IC) is a core executive function that enables us to resist habits, temptations, or distractions (Houdé, 2000; Miyake et al., 2000; Davidson et al., 2006; Diamond et al., 2007). The efficiency of executive functions in childhood, and in particular IC, are a better predictor than socioeconomic status or intelligence (IQ) of later academic success (Moffitt et al., 2011) and health (Diamond, 2013).

Converging evidence suggests that differences in cognitive ability partly trace back to prenatal processes. Indeed, several studies report that subtle variations of the *in utero* environment, as indexed by birth weight, are accompanied by differences in postnatal cognitive abilities (Shenkin et al., 2004; Raznahan et al., 2012; Walhovd et al., 2012). In addition to such a global proxy measure of “uterine optimality” (Raznahan et al., 2012), sulcal patterns, a qualitative characteristic of cortical anatomy, have been used to provide information on the early constraints imposed by the structure of some specific brain regions on later cognitive development (Mangin et al., 2010). Indeed, unlike quantitative features of cortical anatomy (e.g., thickness, surface area), which can take decades to attain the levels observed in adulthood (Giedd and Rapoport, 2010; Raznahan et al., 2011; Li et al., 2014), the qualitative pattern formed by the characteristic set of primary, secondary, and tertiary folds, or sulci, is determined during fetal life and is stable throughout development (Chi et al., 1977; Cachia et al., 2016).

Several studies investigated the long-term influence of normal variation in fetal life on later IC efficiency based on analysis of the sulcal pattern of the dorsal anterior cingulate cortex (ACC). Indeed, ACC is a critical region of the executive network and is constantly activated during IC tasks (Bush et al., 2000; Alvarez and Emory, 2006; Petersen and Posner, 2012). In addition, the ACC presents two qualitatively distinct sulcal patterns (Ono et al., 1990) that can be easily and reliably classified with structural magnetic resonance imaging (MRI; Paus et al., 1996). An asymmetrical sulcal pattern of the ACC was found to be associated with higher IC efficiency in children at age 5 (Cachia et al., 2014) and 9 (Borst et al., 2014) as well as in adults (Fornito et al., 2004; Huster et al., 2009). However, because the sulcal anatomy is very variable (Ono et al., 1990) and complex to analyze, to date, previous studies have only investigated the effect of a single region of the IC neural network on IC efficiency. It is therefore unknown whether the effect of sulcal anatomy on IC efficiency is region specific. A good candidate to test this hypothesis is the inferior frontal cortex (IFC). Indeed, functional neuroimaging studies have consistently associated IC efficiency with the activity of the IFC from childhood to adulthood (for meta-analysis, see Laird et al., 2005).

In this context, the aim of this study was threefold: (1) to replicate previous findings that the sulcal pattern of the ACC affects IC efficiency; (2) to determine whether the sulcal pattern of the IFC also contributes to IC efficiency; and (3) to investigate whether the effects of the sulcal pattern of the ACC and of the IFC are affected by age. In light of the effect of the ACC sulcal pattern asymmetry on IC efficiency reported in previous studies, we anticipated that asymmetric IFC and ACC sulcal patterns would be associated with higher IC efficiency as measured by performance in the color-word Stroop task. The specificity of the potential effects of the IFC and ACC sulcal pattern on IC efficiency was determined by testing whether the sulcal pattern of a cortical area not related to IC efficiency, i.e., the occipito-temporal cortex (OTC), affected participants’ IC efficiency. Additionally, we investigated whether the effects of the sulcal patterns of the IFC and ACC on IC efficiency varies with age by comparing these effects in children and adults. Finally, given that we used the sulcal pattern of the IFC as a proxy for early cerebral constraints on later IC development, we first investigated whether the IFC sulcal pattern was affected by development in our sample by comparing the frequency distribution of the IFC sulcal pattern in children and adults. We reasoned that if the sulcal pattern truly reflects early regional cerebral constraints on cognitive development, then it should not be affected by age, in line with the stability of the ACC sulcal pattern with age previously reported (Cachia et al., 2016). We further investigated the stability of the IFC sulcal pattern from an independent sample of healthy participants with repeated MRI at different ages.

## Materials and Methods

### Participants

The participants consisted of 19 children (M = 10.5 ± 0.87 years old, age range = [9.46:11.89], 10 males) from a public preschool in a location that will be identified if the article is published and 19 young adults (M = 22.2 ± 2.49 years old, age range = [19.05:26.72], 10 males) from the same area. All subjects were right-handed as determined by the Edinburgh Handedness Inventory (Oldfield, 1971). They had no history of neurologic disease and no cerebral abnormalities. All participants provided written consent, or parental/guardian written consent was obtained, which permitted us to enroll the children in the study. All participants were tested in accordance with the national and international norms that govern the use of human research participants. The ethics committee of CPP Nord-Ouest III, France approved our study.

### Behavioral assessment

The participants’ IC efficiency was assessed using the color-word Stroop task, a classic and broadly used experimental paradigm to measure IC abilities (Stroop, 1935; Macleod, 1991). In this task, participants are asked to name either the color of rectangles (the no-conflict condition) or the color of the ink of printed words that denote colors incongruent with the color of the ink (the conflict condition, e.g., “GREEN” printed in blue). In the conflict condition, participants require IC to resolve the conflict between the task-irrelevant information (the color denoted by the word) and the task-relevant information (the ink color).

In each condition, participants denominated the color of 50 items (split over five columns). We used red, green, blue, and yellow (RGB codes 255;0;0, 0;255;0, 0;0;255, and 255;255;0, respectively) for the colors of the rectangles and for the ink colors. Participants were instructed to perform the task as quickly as possible without errors. Reaction times (RTs) were recorded independently for each of the two conditions. For each participant, we computed the Stroop interference score defined as the difference in RT between the conflict and no-conflict conditions. A higher Stroop interference score revealed a lower IC efficiency.

### MRI acquisition

MRIs were acquired at the Cyceron biomedical imaging platform (Caen, France, www.cyceron.fr) using the SENSE parallel imaging technique and a 3T MRI scanner (Archieva, Philips Medical System) with an eight-channel phase array head coil. Structural images were acquired in the sagittal plane with a 3D ultrafast spoiled gradient echo with magnetization preparation sequence. The acquisition parameters were as follows: repetition time = 20 ms; echo time = 4.6 ms; flip angle = 10°; field of view = 256 × 256 mm; matrix size = 256 × 256; slice thickness = 1 mm (voxel size= 1 × 1 × 1); 1 excitation; 180 slices; 252 multishots. The total running time was 9 min 41 s. The same imaging protocol was applied to children and adults. Before the scans, the children were familiarized with the machine’s noise in a MRI mock scanner and were trained not to move during the acquisitions. To reduce motion, the children passively watched a cartoon on an MRI-compatible screen, which also provides a positive experience (Lemaire et al., 2009).

### MRI analysis

The MRI analysis was performed with BrainVISA 4.2 software (RRID:SCR_007354; http://brainvisa.info) using the Morphologist toolbox (RRID:SCR_013248) with standard parameters. An automated pre-processing step was employed to skull-strip T1 MRIs and to segment the brain tissue. The MRI data were spatially linearly normalized to MNI space to control for age-related global differences in brain size. Only linear transformations were used to avoid potential biases resulting from the shape deformations that may occur during the non-linear warping process. Throughout the cortex, the cortical folds were automatically segmented from the skeleton of the gray matter/cerebrospinal fluid mask. The cortical folds corresponded to the crevasse bottoms of the “landscape,” the altitude of which is defined by its intensity on the MRIs. This procedure provided a constant and strong sulcal surface definition that was not influenced by variations in cortical thickness or the gray matter/white matter contrast (Mangin et al., 2004). We visually examined the images at each processing step and for each MRI. No segmentation errors and no motion artifacts were detected.

### Sulcal pattern classification

**Figure 1.**
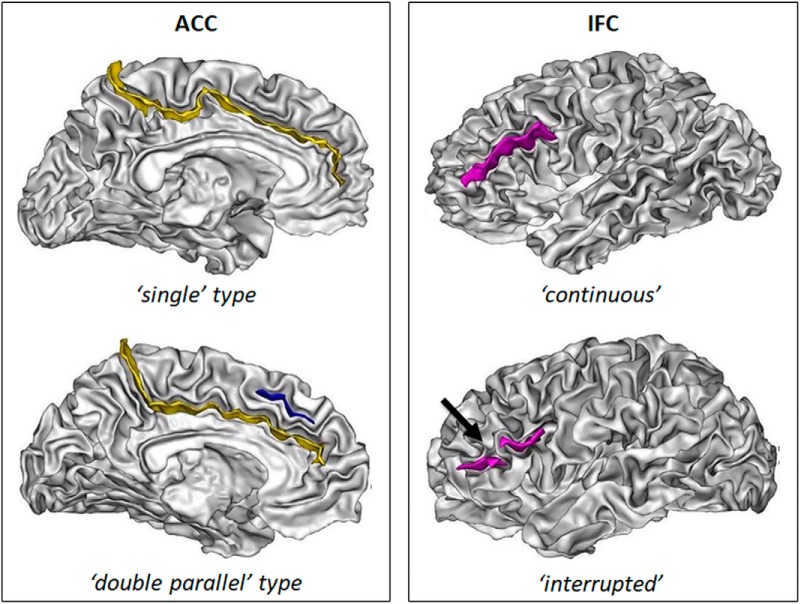
Sulcal patterns of the ACC and the IFC. Left panel, The two ACC sulcal patterns: single type, with only the cingulate sulcus (yellow); and double parallel type, with an additional PCS (blue). Right panel, The two IFC sulcal patterns: with a continuous sulcus or with a sulcus with an interruption (black arrow). The sulci are represented on the cortical surface (gray/white interface).

**Figure 2.**
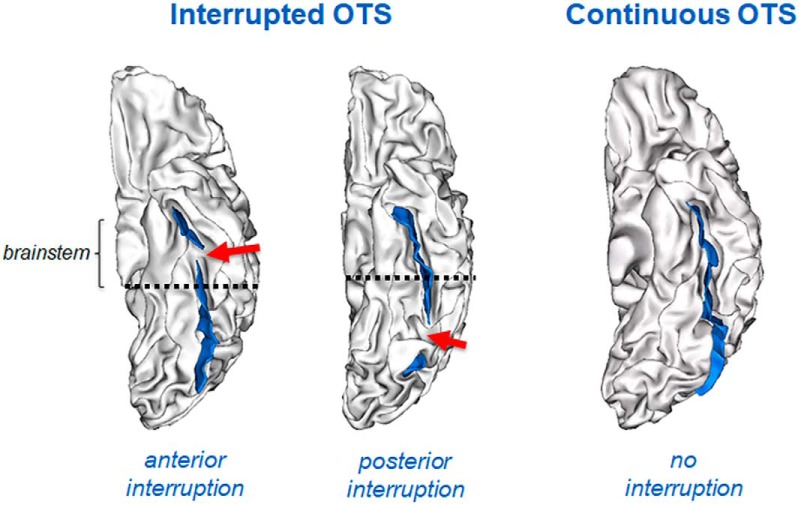
Sulcal patterns of the lateral OTC. Left hemisphere with an anterior interruption (left panel), a posterior interruption (middle panel), or a continuous sulcus. Sulcus are depicted in blue and the sulcal interruption with a red arrow. The PEB (dashed line) was used as a limit to define the anterior and posterior interruptions.

We classified the sulcal patterns of the dorsal part of the ACC and of the IFC (Fig. 1) as well as of the lateral OTC (Fig. 2) in each participant based on Ono’s classification (Ono et al., 1990). The individuals’ 3D mesh-based reconstruction of the cortical folds was visually inspected to classify the sulcal pattern. All MRI data were anonymized, and the ACC and IFC sulcal patterns were independently classified by two of the coauthors (CT, AL). Labeling of the sulcal pattern in a cortical region (ACC, IFC, or OTC) was done blinded to possible confounding information, including the participant’s age and sulcal pattern in another region (ACC, IFC, or OTC). A sulcal pattern was considered “symmetric” when it was identical in both hemispheres and “asymmetric” when it differed across hemispheres.

#### ACC classification

The ACC sulcal pattern was categorized in two types: “single type” or “double parallel type” (Ono et al., 1990) depending on the absence or presence of a paracingulate sulcus (PCS), which is a variable secondary sulcus (Paus et al., 1996; Fig. 1). The PCS was defined as located dorsal to the cingulate sulcus with a course clearly parallel to the cingulate sulcus (Paus et al., 1996; Yücel et al., 2001). To reduce ambiguity from the confluence of the PCS and the cingulate sulcus with the superior rostral sulcus, we determined the anterior limit of the PCS as the point at which the sulcus extends posteriorly from an imaginary vertical line running perpendicular to the line passing through the anterior and posterior commissures (AC-PC; Yücel et al., 2001). The PCS was considered absent if there was no clearly developed horizontal sulcal element parallel to the cingulate sulcus and extending at least 20 mm (interruptions or gaps in the PCS course were not taken into account for the length measure). PCS length was measured in a standard (MNI) space so that the same criterion could be used in children and adults.

#### IFC classification

The IFC sulcal pattern was categorized in two types, “interrupted” and “continuous” (Ono et al., 1990), based on the presence or absence (i.e., continuous sulcus) of an interruption of the inferior frontal sulcus (Fig. 1). Identification of the inferior frontal sulcus was based on Destrieux’s practical guide for the identification of sulcogyral structures (Destrieux et al., 2016). Briefly, the inferior frontal sulcus is a horizontal sulcus running dorsal to the anterior segment of the lateral fissure. Dorsally, the inferior frontal sulcus limits the inferior frontal gyrus, and posteriorly, it connects at a right angle to the inferior segment of the precentral sulcus. After a horizontal course, the inferior frontal sulcus anteriorly takes a descending and then more or less posterior direction. The inferior frontal sulcus may be connected to the precentral sulcus.

#### OTC classification

The lateral OTC was categorized in two types, interrupted and continuous (Ono et al., 1990), based on the presence or absence (i.e., continuous sulcus) of an interruption of the occipito-temporal sulcus (Ono et al., 1990; Borst et al., 2016; Cachia et al., 2017). We distinguished anterior and posterior OTC interruptions (Cachia et al., 2017), with “posterior interruption” corresponding to an interruption located in the posterior part of the sulcus, hosting the visual word form area (VWFA; Vinckier et al., 2007; Dehaene and Cohen, 2011) and the “anterior” interruption corresponded to the anterior part of the sulcus (Fig. 2). We a priori focused on the anterior part of OTC since the posterior part of the left OTC hosting the VWFA, but not the anterior part of the left OTC, contributes to reading skills (Cachia et al., 2017), skills which are involved in the Stroop task used to evaluate IC efficiency. We used an anatomic criterion, namely the *y*-coordinate of the posterior extremity of the brainstem (PEB), as a limit to define the anterior and posterior interruptions of the left and right OTC. The functional validity of this anatomic criterion, which can accurately and reliably demarcate the VWFA in the left OTC, was previously established (Cachia et al., 2017). In addition, such anatomic criterion can be similarly used in the left and right OTC, a critical issue to evaluate the left-right asymmetry of OTC sulcal pattern.

### Longitudinal stability of the IFC sulcal pattern

The stability of the sulcal pattern of the IFC from childhood to adulthood was directly tested using a longitudinal design based on an independent sample with multiple individual MRI scans acquired at different ages. A total of 50 MRI scans of twelve healthy participants (age range: 8.2-27.8 years old; age at first scan: 8.2–11.7 years old; age at latest scan: 15.4–27.8 years old) were selected from a prospective longitudinal study on brain development at the National Institute of Mental Health (NIMH); details on participant recruitment and MRI acquisition can be found in (Giedd et al., 1999). Participants were selected based on the following criteria: (1) the first MRI scan should be acquired before 10 years old; (2) there should be at least three longitudinal MRI scans for the participant; and (3) there should be no gross sulcal segmentation artifact in both left and right prefrontal cortices. The individual IFC sulcal patterns were then randomly and separately classified for each MRI scan. Classification of IFC sulcal pattern was done blinded to possible confounds, including the label of the IFC sulcal pattern in the contralateral hemisphere and in the other time points.

### Statistical analysis

To determine whether the sulcal symmetry of the IFC and ACC was associated with IC efficiency as measured by RTs on the Stroop task, we used a linear model with one categorical within-subject factor, the Stroop condition (“conflict” vs “no-conflict”), and four categorical between-subject factors: the IFC sulcal pattern (symmetric vs asymmetric), the ACC sulcal pattern (symmetric vs asymmetric), the age group (“children” vs “adults”), and the gender (“female” vs “male”). Specifically, we implemented the mixed-effect linear model using repeated-measures MANOVA (Fox and Weisberg, 2010) as detailed below (R syntax):

CONDITION = rbind('incongruent','congruent')

idata <- data.frame(CONDITION)

lm.model <- lm(cbind(RT_Stroop_Incongruent, RT_Stroop_Congruent) ∼ (IFC_Asymetry + ACC_Asymetry) * AgeGroup + Sex)

Anova(lm.model, idata=idata, idesign=∼CONDITION)

Analyses a priori included gender as a covariable because gender was previously shown to have a potential effect on sulcal anatomy (Duchesnay et al., 2007). The interaction between ACC sulcal pattern (two categories) and IFC sulcal pattern (two categories) was not entered into the model due to the restricted number of participants in each of the four categories. To evaluate the specificity of the effects of the ACC and IFC sulcal patterns on IC efficiency, the same analyses were performed again but replacing ACC and IFC factors by a factor related to OTC sulcal pattern (symmetric vs asymmetric).

Main effects and interactions in the linear model were probed with *F* tests derived from Pillai’s trace value. A two-tailed *p* < 0.05 was considered statistically significant. The relative importance of each factor in the linear model was estimated using the “lmg” metric, which is also known as “hierarchical partitioning” (Grömping, 2015). Using this approach, we could decompose the total variance (adjusted R^2^) of the Stroop interference score (RT_conflict condition_ – RT_nonconflict condition_) in four independent sources of variability related to the four factors of the model, i.e., ACC asymmetry, IFC asymmetry, sex, and age. Of note, lmg metric has the advantage to provide a robust estimation of the part of variance explained by each factor, while controlling for possible shared variance with other factors in the model (Grömping, 2015). The 95% confidence intervals (CIs) for relative importance was estimated using 1000 bootstrap replicates. All the statistical analyses were conducted with R 2.9 software (http://www.r-project.org/; RRID = SCR_001905) and “car,” “effects,” “nnet,” “multcomp,” “lattice,” “relaimpo,” “pwr,” and “heplots” libraries.

## Results

### Age effect on the frequency distribution of ACC and IFC sulcal patterns

As anticipated, there was no frequency distribution difference between children and adults in the sulcal patterns of the left and right ACC or in the sulcal patterns of the left and right IFC (Table 1). The observed power value of the statistical tests is reported in Table 2.

**Table 1.** Frequency distribution of the sulcal patterns of the ACC and the IFC in children and adults

			Children (*N* = 19)	Adults (*N* = 19)	Total (*N* = 38)	Children vs adults
ACC	Left	Single	3	4	7	χ = 0.17; *p^a^* = 0.67
		Double parallel	16	15	31	
	Right	Single	9	13	22	χ = 1.72; *p^b^* = 0.19
		Double parallel	10	6	16	
	Asymmetry	Symmetry	7	2	9	χ = 3.64; *p^c^* = 0.06
		Asymmetry	12	17	29	
IFC	Left	Interrupted	7	3	10	χ = 2.17; *p^d^* = 0.14
		Continuous	12	16	28	
	Right	Interrupted	5	4	9	χ = 0.14; *p^e^* = 0.70
		Continuous	14	15	29	
	Asymmetry	Symmetry	11	16	27	χ = 3.19; *p^f^* = 0.07
		Asymmetry	8	3	11	

**Table 2.** Statistical table

Analyses	Variable	Test	Data structure	Type of test	Power
ACC distribution in children and adults	Left pattern	a	Binomial	χ^2^ test	0.07
	Right pattern	b	Binomial	χ^2^ test	0.26
	Pattern asymmetry	c	Binomial	χ^2^ test	0.33
IFC distribution in children and adults	Left pattern	d	Binomial	χ^2^ test	0.31
	Right pattern	e	Binomial	χ^2^ test	0.07
	Pattern asymmetry	f	Binomial	χ^2^ test	0.29
Correlation IFC and ACC	Pattern asymmetry	g	Binomial	χ^2^ test	0.08
Stroop RT and IFC and ACC	Condition: conflict vs non-conflict	h	Normal	*F* test	0.99
	Age: children vs adults	i	Normal	*F* test	0.99
	Interaction: condition × age	j	Normal	*F* test	0.99
	IFC: asymmetry vs symmetry	k	Normal	*F* test	0.18
	ACC: asymmetry vs symmetry	l	Normal	*F* test	0.25
	Interaction: IFC asymmetry × condition	m	Normal	*F* test	0.57
	Interaction: ACC asymmetry × condition	n	Normal	*F* test	0.51
	Interaction: ACC asymmetry × age × condition	o	Normal	*F* test	0.15
	Interaction: IFC asymmetry × age × condition	p	Normal	*F* test	0.05
Stroop RT and Complexity	Complexity	q	Normal	*F* test	0.15
	Interaction: complexity × age	r	Normal	*F* test	0.15
	Interaction: complexity × condition	s	Normal	*F* test	0.22
	Interaction: complexity × condition × age	t	Normal	*F* test	0.05
Stroop RT and OTC	Interaction: OTC asymmetry × condition	u	Normal	*F* test	0.17

**Figure 3.**
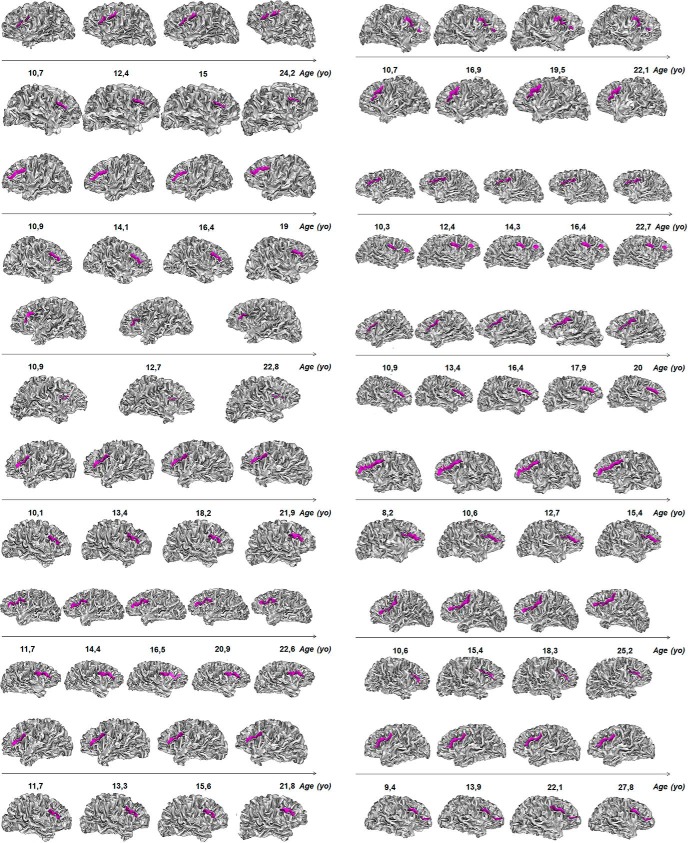
Longitudinal stability of the sulcal pattern (continuous or interrupted) of the IFC (in purple) in the left and right hemispheres.

The complementary analysis of the NIMH sample of healthy participants with repeated scan acquisitions at different ages revealed that the IFC sulcal pattern in the left and right hemispheres remained, without exception, the same at each time point (Fig. 3). The longitudinal stability of the left and right, as well as the left-right asymmetry, of the IFC sulcal pattern was therefore of 100%.

In addition, ACC sulcal pattern symmetry was not correlated with IFC sulcal pattern symmetry, χ^2^(1) = 0.259, *p^g^* = 0.61.

### Effects of sulcal pattern variability on IC efficiency

The analysis of the RTs, based on the linear model with as within subject factor the Stroop condition and as between-factors the IFC and ACC sulcal pattern as well as age group and gender, revealed a classic color-word Stroop effect, with the participants being slower in the conflict (*M* = 54.1 ± 19.1 s) than in the no-conflict (*M* = 30.8 ± 7.8 s) condition, *F*_(1,33)_ = 388.02, *p^h^* < 2.2 × 10^−16^, η^2^_p_ = 0.92. The children (*M* = 52.6 ± 20.3 s) were overall slower than the adults (*M* = 32.3 ± 9.0 s), *F*_(1,33)_ = 46.50, *p^i^* = 8.8 × 10^−8^, η^2^_p_ = 0.58. The main effect of gender was not significant, *F*_(1,33)_ < 1. We found a significant interaction between Stroop condition and age group, with a larger difference in RTs between the conflict and the no-conflict conditions for children (*M* = 69.0 ± 15.2 s and *M* = 36.3 ± 6.8 s, respectively) than for adults (*M* = 39.1 ± 7.1 s and *M* = 25.4 ± 4.0 s, respectively), *F*_(1,33)_ = 52.51, *p^j^* = 2.6 × 10^−8^, η^2^_p_ = 0.61. The interaction between Stroop condition and gender was not significant, *F*_(1,33)_ < 1.

**Figure 4.**
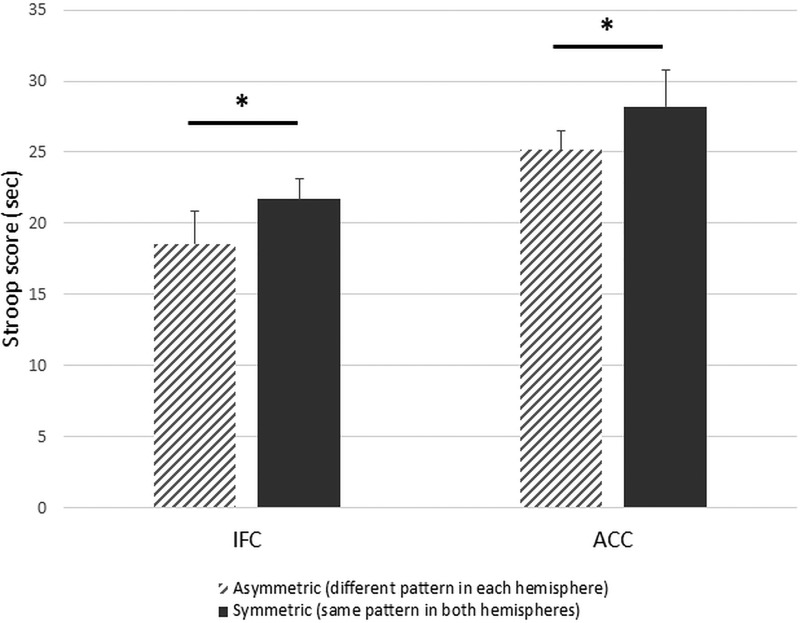
Inhibitory control efficiency and asymmetry of the IFC and ACC sulcal patterns. RTs of Stroop interference scores in participants with a symmetrical sulcal pattern (same pattern in both hemispheres; dark gray) or an asymmetrical sulcal pattern (different pattern in each hemisphere; hatched gray). Error bars denote the SEM. Data were linearly adjusted based on age and gender; *p < 0.05.

**Figure 5.**
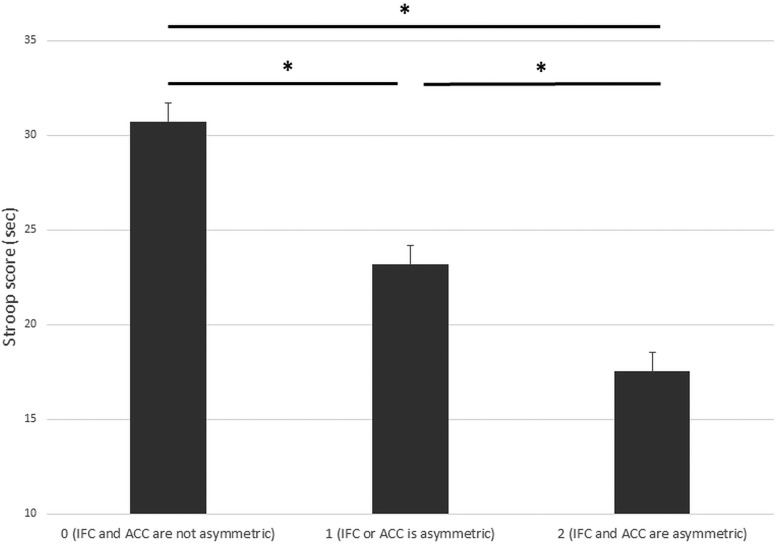
IC efficiency and global asymmetry of the sulcal patterns. RTs of Stroop interference scores in participants with non-asymmetric sulcal pattern (0: IFC and ACC sulcal patterns are not asymmetric), one asymmetric sulcal pattern (1: IFC or ACC sulcal patterns are asymmetric), or two asymmetric sulcal pattern (2: IFC and ACC sulcal patterns are asymmetric). Error bars denote the SEM. Data were linearly adjusted based on age and gender; *p < 0.05.

The main effects of IFC sulcal pattern symmetry, *F*_(1,33)_ = 1.22, *p^k^* = 0.28, and ACC sulcal pattern symmetry, *F*_(1,33)_ = 1.78, *p*^l^ = 0.19, were not significant. However, as expected, the difference in RTs between the conflict and the no-conflict conditions (i.e., the Stroop effect) was greater for participants with symmetric IFC sulcal patterns (*M* = 53.5 ± 20.8 s and *M* = 30.0 ± 8.4 s, respectively) than for participants with asymmetric IFC sulcal patterns (*M* = 55.4 ± 15.2 s and *M* = 33.0 ± 5.5 s, respectively), *F*_(1,33)_ = 5.58, *p^m^* = 0.024, η^2^_p_ = 0.14 (Fig. 4). Similarly, the difference in RTs between the conflict and the no-conflict conditions was greater for participants with symmetric ACC sulcal patterns (*M* = 68.4 ± 23.0 s and *M* = 34.6 ± 10.3 s, respectively) than for participants with asymmetric ACC sulcal patterns (*M* = 49.6 ± 15.7 s and *M* = 29.7 ± 6.6 s, respectively), *F*_(1,33)_ = 4.69, *p^n^* = 0.038, η^2^_p_ = 0.12 (Fig. 4). IFC and ACC sulcal pattern asymmetry explained 3.0% (95% CI = [0.9–10.5]) and 13.7% ([1.5–31.5]), respectively, of the variance in Stroop interference scores (for the cumulative effect of IFC and ACC sulcal pattern asymmetry, see Fig. 5).

We found no triple interaction between Stroop condition, age group and ACC sulcal pattern asymmetry, *F*_(1,31)_ < 1, *p^o^* = 0.36, η^2^_p_ = 0.02, or between Stroop condition, age group and IFC pattern asymmetry, *F*_(1,31)_ < 1, *p^p^* = 0.88, η^2^_p_ = 0.0007, suggesting that the effect of ACC and IFC sulcal pattern asymmetry on the difference in RTs between the conflict and the no-conflict conditions in the color-word Stroop task was similar in children and adults.

Because double parallel ACC and interrupted IFC sulcal patterns represent higher complexity than single ACC and continuous IFC sulcal patterns, we tested the hypothesis that sulcal pattern complexity may be an alternative explanation to sulcal pattern asymmetry. To determine whether complexity may also explain IC efficiency, we tested a complementary model adding a numeric between-subject covariate related to the sulcal complexity, ranging from 0 (single ACC and continuous IFC sulcal pattern) to 4 (double parallel ACC and interrupted IFC sulcal pattern), into the initial model. The main effect of complexity was found to be not significant (*F*_(1,29)_ = 1.16, *p^q^* = 0.29, η^2^_p_ = 0.03), there was no interaction between complexity and age groups (*F*_(1,29)_ = 1.03, *p^r^* = 0.32, η^2^_p_ = 0.03), no interaction between complexity and Stroop condition (*F*_(1,29)_ = 1.73, p^s^ = 0.19, η^2^_p_ = 0.05) and no triple interaction between complexity, Stroop condition and age group (*F*_(1,29)_ = 0.00, *p^t^* = 0.95, η^2^_p_ = 0.0001).

Finally, analysis of the sulcal pattern of the anterior part of the OTC, a cortical area not related to IC efficiency, indicated that, as expected, the interaction between Stroop condition and asymmetry of the OTC sulcal pattern was not significant (*F*_(1,33)_ = 0.00, *p^u^* = 0.99). Furthermore, OTC sulcal pattern asymmetry was found to explain only 0.08% ([0.08–9.7]) of the variance in Stroop interference scores.

## Discussion

Our study provides the first evidence that the sulcal patterns of two regions of the IC network, namely, the dorsal ACC and the IFC, affect IC efficiency. Interestingly, we found the same effect of the ACC and IFC sulcal patterns on IC in children and adults, in line with the notion that the sulcal pattern is an anatomic trait marker of cognition (Borst et al., 2016; Cachia et al., 2016). Our findings replicate previous studies of ACC sulcal patterns and IC efficiency (Fornito et al., 2004; Huster et al., 2009; Borst et al., 2014; Cachia et al., 2014) and extend them to another cortical region, namely, the IFG. Finally, we found that the frequency distribution of the sulcal patterns of the ACC and IFC was similar across ages. Because there were trends toward differences in sulcal pattern asymmetry between adults and children, we performed a complementary analysis to confirm the stability of IFC sulcal pattern during development. Using a longitudinal design from an independent sample of healthy participants with repeated scan acquisitions at different ages, we found that individual sulcal patterns of the left and the right IFC remain stable during development. These results are consistent with a previous longitudinal study showing stability of the ACC sulcal pattern throughout development (Cachia et al., 2016). Taken together, such findings provide further evidence that interindividual differences in IC efficiency partly arise from the fetal stages of brain development, when the sulcal patterns of the ACC and IFC are determined (Chi et al., 1977; Cachia et al., 2016) under the effects of genetic and environmental factors (Dehay et al., 1996; Molko et al., 2003; Rakic, 2004; White et al., 2010; Barkovich et al., 2012). Such analysis of long-term effect of fetal life on later IC efficiency complement findings from previous studies suggesting that individual differences in IC efficiency were related to individual differences in the volume, surface or cortical thickness of the ACC and of the IFC (Casey et al., 1997; Tamnes et al., 2010; Fjell et al., 2012; Takeuchi et al., 2012; Kharitonova et al., 2013), which constitute typical markers of neuroplasticity (Zatorre et al., 2012). The precise mechanism underlying cortical folding is still unknown. However, several factors likely contribute to the prenatal processes that influence the shape of the folded cerebral cortex, including cortical growth (Kuida et al., 1996; Haydar et al., 1999; Chenn and Walsh, 2002; Toro and Burnod, 2005), apoptosis (i.e., programmed cell death; Haydar et al., 1999), differential expansion of superior and inferior cortical layers (Richman et al., 1975; Kriegstein et al., 2006), differential tangential expansion (Ronan et al., 2014), and/or structural connectivity through axonal tension forces (Dehay et al., 1996; Hilgetag and Barbas, 2006; Van Essen, 1997).

The lack of correlation between the frequency distribution of IFC and ACC sulcal patterns might be associated with the specific functional role of the ACC (Ebitz and Hayden, 2016; Kolling et al., 2016; Shenhav et al., 2016) and the IFG (Aron et al., 2014; Swick and Chatham, 2014) in IC. The ACC is classically associated with cognitive control (Shackman et al., 2011; Petersen and Posner, 2012; Shenhav et al., 2013), including conflict monitoring (Carter et al., 1998; Botvinick et al., 1999, 2001, 2004; van Veen et al., 2001; Kerns et al., 2004). During the Stroop task, the ACC can be viewed as a central executive system (Peterson et al., 1999) that monitors ongoing processing and signals conflict between potential responses and indicates the need for additional cognitive resources to the cognitive control system sustained by the dorsolateral prefrontal cortex (Egner and Hirsch, 2005). A recent integrative theory proposes that ACC activity is involved not only in cognitive control but also in evaluation and motivation (Shenhav et al., 2013, 2016). Such a model helps specify the currently optimal allocation of control by establishing the overall expected value of control. Several functional roles have been attributed to the IFG. For instance, the IFC has been associated with selective attention processes (Kemmotsu et al., 2005), controlling competing responses and refocusing attention on relevant stimulus features (Zysset et al., 2001). The IFC, and particularly the inferior frontal junction, might also be critical for updating task representations (Brass et al., 2005; Derrfuss et al., 2005). During motor response inhibition, Aron et al. (2004, 2014) considered the right IFC as a brake that can be activated in different modes (i.e., totally, to stop a response; or partially, to pause) and in different contexts (i.e., externally, by stop or noticeable signals; or internally, by goals). Subregions of the right IFC might also be involved in a broad but distinct aspect of task-oriented processing (Hampshire et al., 2010, 2012; Hampshire, 2015). This study focused on a single cognitive domain (IC) assessed with a single test (Stroop task). It was therefore not possible to directly test the specificity of the observed relationship between ACC and IFC sulcal patterns and IC efficiency. It is important to note, however, that previous studies have provided evidence that the effects of the ACC sulcal patterns were specific to IC efficiency (Borst et al., 2016; Cachia et al., 2016). That said, we found that the sulcal pattern of the anterior part of the OTC, a cortical area not directly involved in IC, did not affect participants’ IC efficiency in the present study providing evidence in favor of the specificity of the effects of the sulcal pattern of the ACC and IFC on IC efficiency. Finally, we note that it would be interesting to investigate whether the sulcal pattern of other prefrontal brain regions involved in IC also contribute to IC efficiency. A good candidate might be the orbitofrontal cortex (OFC) essentially because its sulcal pattern variants are clearly documented (Nakamura et al., 2007). However, we could not directly test the possible effect of OFC sulcal pattern on IC efficiency in the current study because the OFC segmentation and the 3D mesh-based rendering was not good enough, likely because of acquisition artifacts related to MRI signal decrease due to sinus air/bone interface, to reliably distinguish the different OFC sulcal patterns, i.e., connection, or not, of the medial and lateral orbital sulci.

Our finding of additive effects of IFC and ACC sulcal patterns on IC efficiency suggests that distinct early neurodevelopmental mechanisms targeting different brain regions likely contribute to IC efficiency. This interpretation is in line with a recent study of schizophrenia showing that IC variability may be the final common pathway of several early neurodevelopmental mechanisms (Gay et al., 2016). This view shares some analogies with the “common variant–small effect” model in genetics (Bodmer and Bonilla, 2008). This classic genetic model of complex and multifactorial conditions (e.g., psychiatric or cardiovascular disorders) states that frequent genetic polymorphisms (e.g., single-nucleotide polymorphisms; SNP) have small effects but collectively account for a large portion of the variance. Similarly, each sulcal polymorphism has a small but additive effect: the IFG and ACC sulcal patterns explain, respectively, around 3% and 14% of the variance of the Stroop interference scores. The large CIs for the relative importance of IFG and ACC are likely related to the relatively small sample size. Of note, the lower bond of the CI related to IFG was strictly >0, thus providing evidence that the contribution of IFG while being limited is not null. In addition, it is important to note that the sulcal pattern in a cortical area not directly involved in IC (i.e., the anterior part of the OTC) contribute to 0.08% of the variance of IC versus 3% of the variance explained by the sulcal pattern of the IFC. Although analogy has well-known limitations (Hofstadter and Sander, 2013), it allows for the generation of original models derived from one domain and translated to another domain. Following this analogy, some properties of the genetic polymorphisms could be translated to the sulcal polymorphisms. For instance, the genetic notion that human cells have two homologous copies/alleles of each gene (diploidy) is very close to the neural notion that the cortical sulci have two homologous copies in each hemisphere. Genetic zygosity and heterozygosity (same or different gene alleles) is therefore analogous to sulcal symmetry and asymmetry (same or different sulcal pattern in left and right hemispheres). In addition, the recent finding of a specific abnormal sulcal pattern in the central/precentral region in type 2 focal cortical dysplasia (Mellerio et al., 2015) suggests that in addition to the common variant–small effect model, the “rare variant–high effect” genetic model may also be relevant for understanding sulcal polymorphisms.

Although our findings are suggestive of a causal role of sulcation in determining later IC efficiency, a direct causal link has yet to be established. A longitudinal study with long-term follow-up from birth, or young age, to adulthood, a period of major IC efficiency change (Luna et al., 2004; Luna, 2009), could provide such evidence. It would also be informative to follow individual children (for review, see Diamond, 2013) or adults (Diamond, 2013; Maraver et al., 2016; Zhao et al., 2016) during intense short-term training of IC and to investigate the possible modulatory effect of the ACC and IFC sulcal patterns on the receptivity to IC training, namely whether cognitive changes after IC training are different, or not, in participants with different ACC and IFC sulcal patterns. The sulcal patterns is a feature robust to neuroplastic processes underlying brain development (Chi et al., 1977; Cachia et al., 2016) and thus should not be modified by IC training. This interpretation is consistent with the findings that the sulcal pattern of the left lateral OTC (Cachia et al., 2017), a cortical region hosting the VWFA and associated with reading skills in children (Borst et al., 2016) and adults (Cachia et al., 2017) is not affected by learning to read which typically requires an intense and protracted cognitive training.

In addition, the physiologic mechanisms underlying the association of IC efficiency and sulcal pattern asymmetry are not straightforward. This association is likely mediated by the effect of sulcal pattern on the functional brain activity (Crosson et al., 1999; Artiges et al., 2006; Amiez et al., 2013). For instance, functional MRI activation during an interference task was found to be left-sided in subjects with double-parallel type ACC, and right-sided in subjects with single type ACC (Artiges et al., 2006). Several studies have also reported a correlation between the shape of the folded cerebral cortex and the underlying structural connectivity through axonal tension forces (Dehay et al., 1996; Van Essen, 1997; Hilgetag and Barbas, 2006; Leonard et al., 2009). Therefore, we speculate that the differences in IC efficiency observed in children and adults with symmetrical versus asymmetrical sulcal patterns might be associated with differences in brain network efficiency due to differences in interhemispheric brain connectivity. Increased cognitive efficiency in asymmetric brains might be associated with hemispheric specialization, as it is more efficient to transfer information between close areas within the same hemisphere than between distant areas distributed across the two hemispheres (Toga and Thompson, 2003; Deary et al., 2010). In addition, asymmetry might enhance the specialization of neural substrates by limiting useless replication of identical circuits in both hemispheres (Levy, 1977) and decrease conflict between the two hemispheres (Concha et al., 2012). Such an association between hemispheric specialization and brain asymmetry is supported by anatomic studies of the corpus callosum (i.e., a large bundle of interhemispheric fibers) that showed that asymmetrical brains have fewer and/or thinner fibers connecting the two hemispheres than more symmetrical brains, as revealed by a reduced midsagittal area (Witelson, 1985) and microstructural integrity measured using diffusion MRI (Putnam et al., 2008). Individuals with no corpus callosum (i.e., complete agenesis) exhibit an intact Stroop interference effect (Brown et al., 2001), suggesting that the processes involved in performing the Stroop task are highly lateralized in the brain. In this context, further investigation of the influence of the lateralization of the sulcal pattern (i.e., whether double parallel ACC or interrupted IFC is present in the right or left hemisphere in asymmetric sulcal patterns) on IC efficiency on large sample could provide interesting insights. Such analysis could not be performed in the current study because the unbalanced distribution of leftward and rightward asymmetries led to a very limited number of participants in some categories (e.g., *N* = 1 adult with rightward IFC asymmetry, *N* = 2 adults with leftward IFC asymmetry, *N* = 3 children with rightward ACC asymmetry). Finally, an alternative interpretation of the increased IC efficiency found in participant with asymmetric sulcal patterns relies on the increased complexity of asymmetric sulcal patterns. Indeed, although the formal definition of sulcal pattern complexity is not straightforward, one can argue that double parallel ACC, or interrupted IFC, represent higher complexity, in terms of increased number of folds, than single ACC, or continuous IFC. This alternative hypothesis was ruled out in the present study as suggested by the lack of significant effect of the complexity of the sulcal pattern of these two cortical areas on IC efficiency. However, it is important to note that the values of the complexity covariate were not equally distributed (17 participants with a complexity of 1, 15 with a complexity of 2, five with a complexity of 3, and one with a complexity of 4) and may have biased and/or limited the power of the statistical analysis which should therefore be replicated in a larger sample for confirmation.

In conclusion, this study provides the first evidence that sulcal polymorphisms in the ACC and the IFG complementarily contribute to variability in IC efficiency in children and adults, suggesting that IC variability may be the final common pathway of several early neurodevelopmental mechanisms targeting different cortical areas.

## Synthesis

Reviewing Editor: Philippe Tobler, University of Zurich Decisions are customarily a result of the Reviewing Editor and the peer reviewers coming together and discussing their recommendations until a consensus is reached. When revisions are invited, a fact-based synthesis statement explaining their decision and outlining what is needed to prepare a revision will be listed below.

Thank you for a responsive revision.

One minor point remains (concerning previous intermediate point 1):

In order to prevent confusion between figures 4/5 and text results, please provide also the adjusted numbers in the text.
